# Serum neuron-specific enolase, magnetic resonance imaging, and electrophysiology for predicting neurodevelopmental outcomes of neonates with hypoxic-ischemic encephalopathy: a prospective study

**DOI:** 10.1186/s12887-022-03329-8

**Published:** 2022-05-17

**Authors:** Hui-Zhi Huang, Xiao-Feng Hu, Xiao-Hong Wen, Li-Qi Yang

**Affiliations:** 1 Department of Pediatrics, The Second Affiliated Hospital of Anhui Medical University, Hefei, Anhui China; 2 Department of Neonatology, Anhui Provincial Children’s Hospital/Children’s Hospital of Anhui Medical University, Hefei, Anhui China; 3 Department of Radiology, The First Affiliated Hospital of USTC, Division of Life Sciences and Medicine, University of Science and Technology of China, Hefei, Anhui China; 4 Department of Pediatrics, The Third Affiliated Hospital of Anhui Medical University, Hefei, Anhui China

**Keywords:** Newborn, Hypoxic-ischemic encephalopathy, Prognosis, Neuron-specific enolase, Magnetic resonance imaging, Electroencephalography

## Abstract

**Background:**

Neonatal hypoxic-ischemic encephalopathy (HIE) is an important cause of mortality and morbidity. Effective indicators for the early diagnosis of brain injury after HIE and prognosis are lacking. This study aimed to examine the predictive value of serum neuron-specific enolase (NSE), amplitude-integrated electroencephalography (aEEG), and magnetic resonance imaging (MRI), alone and in combination, for the neurological outcomes in neonates with HIE.

**Methods:**

Newborns with HIE born and treated at the Third Affiliated Hospital of An-Hui Medical University were consecutively included in this prospective cohort study (June 2013 to December 2020). Encephalopathy was classified as mild, moderate or severe according to Samat and Sarnat. All patients were assessed serum 1-day NSE and 3-day NSE levels after birth. The children were classified by neurological examination and Bayley Scales of Infant Development II at 18 months of age. ROC analysis was used to evaluate the predictive accuracy of the neurodevelopment outcomes.

**Results:**

A total of 50 HIE neonates were enrolled (normal group: 32 (64.0%), moderate delay: 5 (10.0%), severe delay: 30(26.0%)) according to Bayley II scores. Serum 3-day NSE levels increased with worsening neurodevelopment outcomes (normal: 20.52 ± 6.42 μg/L vs. moderate: 39.82 ± 5.92 μg/L vs. severe: 44.60 ± 9.01 μg/L, *P* < 0.001). The MRI findings at 4–7 days after birth were significantly different among the three groups (*P* < 0.001). Forty-two (84.0%) children had abnormal aEEG. The combination of the three abnormalities combined together had 100% sensitivity, 97.70% specificity, 98.25% PPV, and 99.98% NPV.

**Conclusions:**

MRI, aEEG, and 3-day NSE can predict the neurological prognosis of newborns with HIE without hypothermia treatment. Their combination can improve the predictive ability for long-term neurobehavioral prognosis.

## Background

Neonatal hypoxic-ischemic encephalopathy (HIE) is a type of encephalopathy caused by oxygen deprivation to the brain [1, 2]. It is mainly reported and studied in infants > 35 weeks gestation [1, 2]. Of note, the definition of HIE is more difficult in preterm infants, and preterm infants are not eligible for cooling; therefore, they have not been studied thoroughly. The reported incidence of neonatal HIE is 2–8 per 1000 live births in developed countries and 26 per 1000 live births in underdeveloped countries [2–4]. It is most likely the result of an acute peripartum (most common) or chronic (during pregnancy) event [1, 2]. It can be caused by sentinel hypoxic or ischemic events such as severe placental abruption, umbilical cord prolapse, or shoulder dystocia, but most cases are idiopathic [1, 4]. Common neurologic sequelae of HIE include neurodevelopmental disabilities such as cerebral palsy, seizures, hearing loss, blindness, learning disabilities, and behavioral disabilities [1, 2, 5, 6]. Predicting the neurological prognosis would ensure optimal management and treatment and avoiding exposure to potentially toxic therapies [7, 8].

In the last decades, the Apgar score and Sarnat staging have been applied to predict prognosis [4], but follow-up studies demonstrated a low predictive value of Sarnat grading for the outcome of infants with HIE, especially moderate (Sarnat grade II) encephalopathy [9–12]. The infant’s condition after therapeutic hypothermia, including neurophysiologic tests, amplitude-integrated electroencephalography (aEEG), biochemical markers, and neuroimaging, have been used to assess the severity of HIE and predict long-term outcomes [1, 2, 4, 8, 13, 14]. Indeed, the tests above are recommended by guidelines [1] and by the literature [2, 4]. In addition, cranial ultrasound, computed tomography (CT), magnetic resonance imaging (MRI) are valuable tools to determine the extent of ischemia and brain damage [8], with MRI being the most sensitive modality for determining the patterns of brain injury, without the use of radiations [13, 14]. Unfortunately, the results are influenced by the timing of the monitoring among studies [15–17] or the different timing of the different examinations within a given study [15].

Neuron-specific enolase (NSE) is specific to central and peripheral neurons and neuroendocrine cells [18]. High blood levels of NSE are associated with poor outcomes after cardiac arrest, stroke, and pediatric traumatic brain injury [18]. Celtik et al. [19] showed that NSE could be used as a predictor of HIE severity. Hypothermia treatment for HIE might be associated with decreased NSE levels and possibly with neurodevelopmental outcomes [20], but the results need to be confirmed. Still, whether a combination of different methods could improve the prognostication of HIE remains to be examined. Some recent studies examined NSE levels, MRI, and aEEG on the outcomes of HIE within the same study, but without combining the different modalities [21, 22]. A study suggested that the combination of cerebrospinal fluid NSE, MRI, and EEG could predict the outcomes of HIE [23], but sampling cerebrospinal fluid in neonates is riskier than blood.

Therefore, this study aimed to examine the predictive value of serum NSE, aEEG, and MRI, alone and in combination, for the neurological outcomes in neonates with HIE.

## Methods

### Patients

All consecutive surviving full-term newborns with HIE born and treated at the Neonatal Intensive Care Unit of the Third Affiliated Hospital of An-Hui Medical University and subsequently followed at the same hospital were included in this prospective cohort study (June 2013 to December 2020). Ethical approval was obtained from the clinical research and ethics committee of the Third Affiliated Hospital of An-Hui Medical University (#2013–021-01). All parents gave their informed consent.

The inclusion criteria were 1) born after a gestational age of ≥37 and < 42 weeks [24–26], 2) birth weight of 2500–4000 g [24–26], 3) initial umbilical artery blood pH < 7.0, 4) Apgar score 0–3 for more than 5 minutes [27], 4) abnormal neurological manifestation, including included altered consciousness (irritation, drowsiness, or coma), muscle tone (increased or decreased), primal reflexes (decreased or disappeared from sucking or embrace reflexes), convulsive seizures, brainstem symptoms (altered respiratory rhythm, altered pupil, retardation or disappearance of light reflexes), and increased fontanelle tension, and 5) multiple organ dysfunction occurred shortly after birth (two or more organ dysfunction, including cardiovascular, gastrointestinal, hematologic, pulmonary, or renal systems) [27–29]. The exclusion criteria were 1) congenital malformations or major dysmorphic features, 2) congenital viral infections, and 3) defined metabolic syndromes.

Respiratory dysfunction was defined as 1) respiratory rate > 90 breaths/min, 2) PaO_2_ < 5.3 kPa in the absence of cyanotic disease, 3) PaCO_2_ > 8.7 kPa, PaO_2_/FiO_2_ < 200 Torr in the absence of cyanotic disease, or 4) mechanical ventilation [29]. Cardiovascular dysfunction was defined as 1) systolic blood pressure < 40 mmHg, 2) heart rate < 50 or > 220 bpm, 3) cardiac arrest, 4) pH < 7.2 with normal PaCO_2_, or 5) continuous hemodynamic support [29]. Hematologic dysfunction was defined as 1) hemoglobin < 5 g/L, white blood cells < 3 × 10^9^/L, 3) platelet counts < 20 × 10^9^/L, or 4) disseminated intravascular coagulation [29]. Neurologic dysfunction was defined as 1) Glasgow coma score < 5 or 2) fixed dilated pupils [29]. Hepatic dysfunction was defined as total bilirubin > 60 μmol/L [29]. Gastrointestinal dysfunction was defined as 1) upper gastrointestinal bleeding or 2) any of hemoglobin by > 20 g/L, blood transfusion, hypotension <3rd percentile, or gastric or duodenal surgery [29]. Renal dysfunction was defined as 1) blood urea nitrogen > 36 mmol/L, 2) serum creatinine > 177 μmol/L, or 3) dialysis/hemofiltration [29].

Hypothermia was not used in this study, ruling out the influence of hypothermia on many test results. Mild hypothermia is the HIE grade A treatment evidence, but many hospitals in China do not have mild hypothermia treatment equipment. Different from Western countries, hospitals without such treatment equipment will transfer children to hospitals with medical equipment; but in China the medical system is continuing to improve and transport systems are not yet fully developed, such hospitals are rare, often far away, and the children are not transported in time. Still, all patients received appropriate ventilation and perfusion, maintaining blood glucose levels, and controlling convulsions and cerebral edema.

### Amplitude-integrated electroencephalography

According to the hospital protocol after admission, two-channel monitoring of 8 electrodes aEEG (Bio-logic Ceegraphscan Netlink Monitor, NATUS Medical Incorporated, Mundelein, IL, USA) within 6 h after birth was recorded for 4 h [30, 31]. The aEEGs were assessed by two independent researchers blinded to the clinical data. The representative background pattern was determined using the scoring system suggested by Hellstrom-Westas et al. [32, 33]. The aEEG results were divided into three categories according to background activity of the neonatal aEEG: 1) normal amplitude: the boundary of the amplitude spectrum was > 10 μV, and the lower boundary was > 5 μV; 2) mildly abnormal amplitude: boundary > 10 μV and lower boundary ≤5 μV in the spectral band; 3) severe amplitude anomaly: the boundary of the spectral band was < 10 μV, and the lower boundary was ≤5 μV. All three categories might be associated with an epileptic activity. According to aEEG background activity and the presence or absence of epileptic activity, the aEEG results were divided into four categories: 1) normal aEEG with normal amplitude; 2) mildly abnormal aEEG with normal amplitude and epileptic activity; 3) mildly abnormal aEEG with normal amplitude; 4) all other combinations were severely abnormal aEEG. According to the needs of this study, the results were divided into two categories, normal and abnormal (including mild and severe abnormalities).

### Serum NSE levels

NSE was detected at 1 day (1-day NSE) and 3 days (3-day NSE) after birth. NSE was measured on a Cobas Core II Immunoanalyzer with the NSE EIA II kit (Roche Molecular Biochemicals, Mannheim, Germany), a one-step sandwich-type enzyme immunoassay that uses monoclonal mouse antibodies. The manufacturers claim a low detection limit of 0.1 μg/L for NSE.

### Brain MRI

MRI (T1- and T2-weighed imaging) scans were obtained within the 4–7 days after birth [34–36] when the vital sign of the children was relatively stable. All infants were scanned with a 1.5-T Magnetom Avanto MRI system (Siemens, Erlangen, Germany), including MRI plain0 T1WI and T2WI scans. T1WI sequence parameters: RF pulse repetition time 585 ms, echo time 15 ms. T2WI sequence parameters: RF pulse repetition time 4000 ms, echo time 102 ms, a layer thickness of 4 mm, layer spacing of 0.4 mm, and field of view of 20 × 20 cm. All MRIs in this study were read independently by two radiologists who specialized in neuroimaging, and they discussed their findings together. The basal ganglia/watershed scores were determined as proposed by Barkovich et al. [37]: 0 = no abnormalities in the basal ganglia or cortex; 1 = an abnormal signal in the basal ganglia or thalamus; 2 = an abnormal signal in the cortex; 3 = an abnormal signal in the cortex and basal nuclei (basal ganglia or thalami); 4 = an abnormal signal in the entire cortex and basal nuclei. All images and qualitative scores were assessed by an experienced radiologist, who was blinded to the serum NSE levels and clinical data.

### Follow-up and neurodevelopment outcomes

Periodical follow-ups of the neurodevelopment outcomes were performed once a month before 6 months of age, every 2 months between 6 months and 1 year, and every 6 months after 1 year. At 18 months of age, the children were assessed in the neurological rehabilitation clinic according to the study protocol. All children received standardized assessment at 18 months. None of the children were lost to follow-up. If the evaluation could not be made according to the appointment time, telephone communication, or picking up the children at home to the hospital for evaluation, to avoid the loss of follow-up and data loss.

The neurodevelopmental function was assessed with the Bayley Scales of Infant Development 2nd version (BSID-II) [38] and neurological examination by a pediatric neurodevelopmental specialist. Motor testing at 1 year improves the prediction of motor and mental outcomes at 2 years after perinatal HIE [39]. The pediatrist was not involved in the neonatal and postnatal care of the children under investigation and was blinded for the clinical status, histories, NSE levels, and neonatal brain MRI scores of the children. This examination took place at a time chosen by the mother as the most optimal time for the child to cooperate, mostly around 10 a.m. The children were classified into three groups depending on the examination results; normal (MDI and PDI > 79), moderate delay (MDI or PDI: 70–79), and severe delay (MDI or PDI < 70) [40, 41]. When normal and abnormal outcomes were predicted, excepting for MDI and PDI in BSID (both mild and severe abnormalities were classified as abnormal) were used to determine the neurodevelopment outcome, a diagnosis of cerebral palsy, visual impairment and hearing loss or epilepsy was also considered an abnormal outcome.

### Statistical analysis

Statistical analysis was conducted using SPSS 17.0 (SPSS, NY, USA). All values are presented as mean ± standard deviation or median (range). The distributions of the continuous data were assessed using the Shapiro-Wilk tests. ANOVA and Tukey’s HSD post hoc test were used to analyze normally distributed data, while the Kruskal-Wallis test was used for non-parametric analyses. Categorical data are presented as n (%) and were analyzed using the chi-square test. A logistic regression model was fit to the binary outcome using aEEG, NSE, and brain MRI together as predictors for neurodevelopment delay. Receiver operating characteristic (ROC) analysis was used to evaluate the predictive accuracy of the logistic regression model, as well as aEEG, NSE, and brain MRI, separately and together. Sensitivity, specificity, PPV, and NPV of aEEG, NSE, and brain MRI, separately and together, were calculated using binomial exact method. *P*-values < 0.05 were considered statistically significant.

## Results

### Characteristics of the patients

**Fig. 1:**
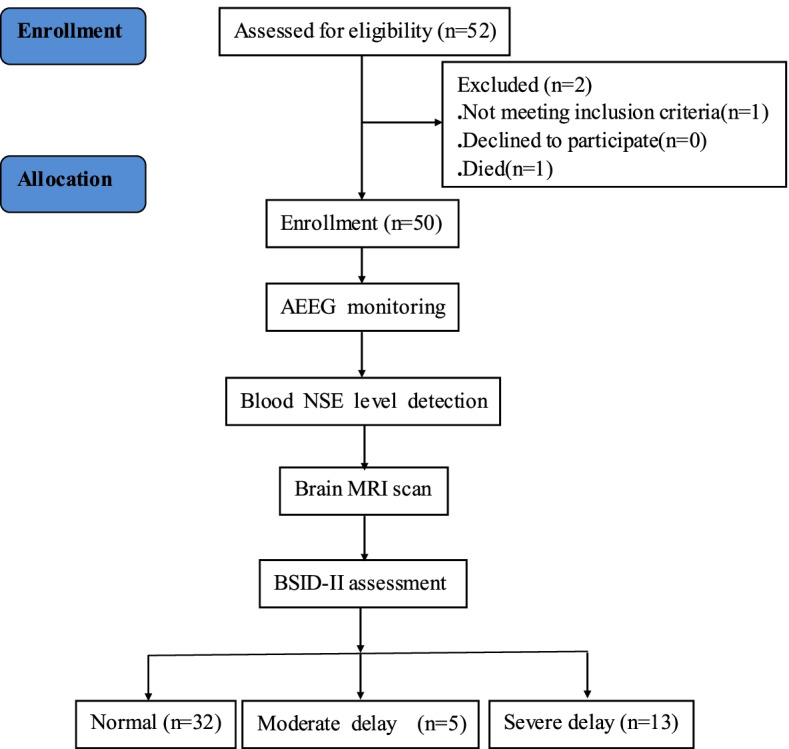
Patient flowchart

A total of 50 HIE neonates were recruited and followed during the study period (Fig. 1). The neonates were divided into three groups based on according to Bayley II scores: 32 children (64.0%) were in the normal group; 5 (10.0%) and 13 (26.0%) children were in the moderate and severe neurodevelopmental delay group, respectively. The characteristics of the study population are shown in Table 1. There were no significant differences in sex, birth weight, gestational age, Apgar scores, and HIE severity among the children with three different neurodevelopment outcomes. Six neonates had recurrent seizures, which were significantly associated with poor prognosis (*P* < 0.001), and 12 had single clinical or subclinical seizures (six had poor outcomes, and six had good outcomes). The antiepileptic drugs used were mainly phenobarbital sodium and midazolam. More infants with severe delay were intubated or ventilated (*P* = 0.046) or received epinephrine (*P* = 0.030) in the delivery room. The BSID-II MDI and PDI scores decreased with increasing severity of delay (both *P* < 0.001) (Table 1).

**Table 1:** Baseline characteristics and outcome data (*n*=50)

Characteristics	Neurodevelopment outcome			*P*
	Normal (*n*=32)	Moderate delay (*n*=5)	Severe delay (*n*=13)	
Sex				0.502
Male	18 (56.2%)	2 (40.0%)	9 (69.2%)	
Female	14 (43.8%)	3 (60.0%)	4 (30.8%)	
Birth weight (g)	3161±397	2910±407	3118±377	0.502
Gestational age (week)	39.6±1.0	39.3±0.7	39.4±0.9	0.688
BSID-II MDI	95.06±9.26	74.20±3.27	64.54±3.59	<0.001
BSID-II PDI	94.84±5.89	74.20±3.49	65.08±4.23	<0.001
HIE severity				0.063
grade I	11 (34.4%)	0 (0%)	4 (30.8%)	
grade II	21 (65.6%)	4 (80.0%)	7 (53.9%)	
grade III	0 (0%)	1 (20.0%)	2 (15.4%)	
Apgar score (5 min), median (IQR)	2 (0~3)	2 (1~3)	2 (0~3)	0.673
pH, median (IQR)	7 (6.81-7.25)	7 (6.80-7.15)	7 (6.81-7.19)	0.005
<7.0, n (%)	13(40.63)	2(40.00)	5(38.46)	0.921
Delivery room resuscitation, n (%)				
Intubation or ventilation	18(56.25)	4(80.00)	12(92.31)	0.046
Chest compression	10(31.25)	3(60.00)	8(61.54)	0.140
Epinephrine administration	4(12.50)	2(40.00)	6(46.15)	0.030
aEEG (<6 h after birth)				<0.001
Normal	8 (25.0%)	0 (0%)	0 (0%)	
Abnormal	24 (75.0%)	5 (100%)	13 (100%)	
1-day NSE	40.31±7.98	41.74±8.34	40.85±8.92	0.929
3-day NSE	20.52±6.42	39.82±5.92	44.60±9.01	<0.001
Brain MRI				<0.001
Grade 0	23 (71.9%)	1 (20.0%)	0 (0%)	
Grade 1	7 (21.9%)	0 (0%)	0 (0%)	
Grade 2	2 (6.3%)	0 (0%)	4 (30.8%)	
Grade 3	0 (0%)	2 (40.0%)	4 (30.8%)	
Grade 4	0 (0%)	2 (40.0%)	5 (38.5%)	
3-7 days neurologic exam	5(15.63)	2(40.00)	10(76.92)	<0.001

Values are expressed as number (%), mean ± SD, or median and range. *HIE* Hypoxic-ischemic encephalopathy. *1-day NSE* Serum NSE level of 1 day after birth, *3-day NSE* Serum NSE level of 3 days after birth. *aEEG* Amplitude-integrated electroencephalography

### aEEG, NSE levels, and MRI of the neonates with HIE

**Fig. 2:**
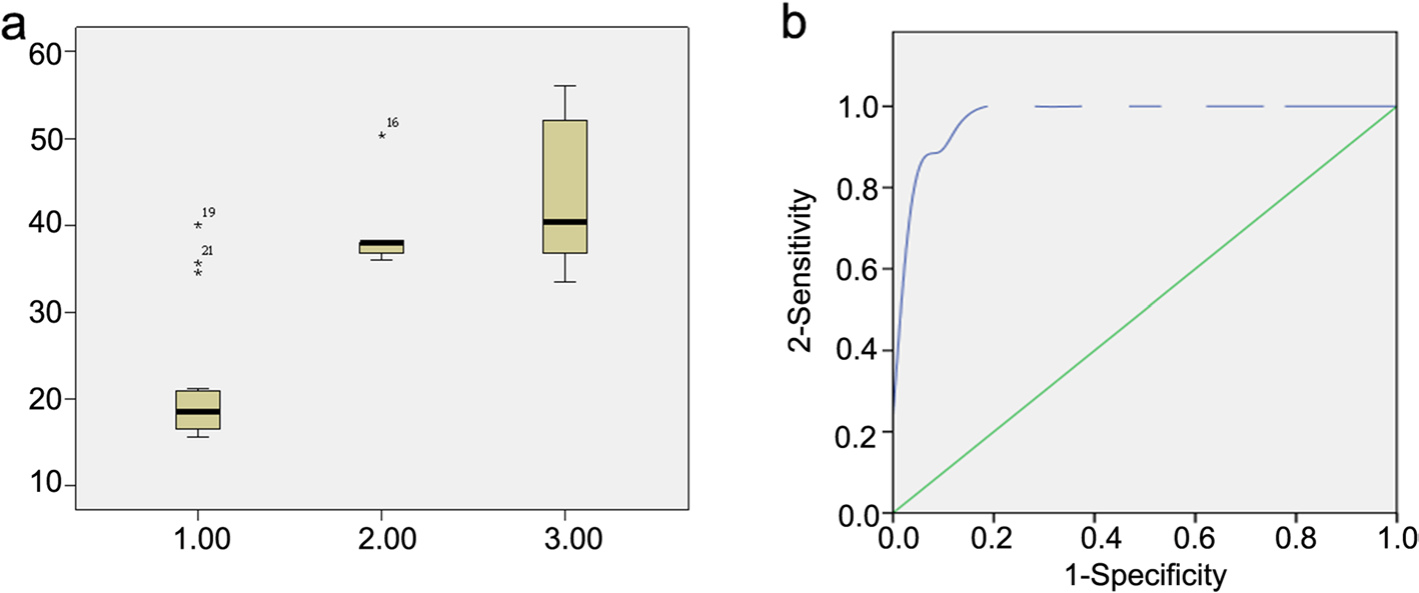
**a** Serum neuron-specific enolase 3-day NSE levels in relation to outcome (1: normal, 2: moderate delay, 3: severe delay). **b** Receiver operating characteristic (ROC) curves for 3-day NSE (cut-off point 27.3 μg/L) as a marker for distinguishing infants with developmental delay from infants with the normal outcome, ROC curves for 3-day NSE, Spearman correlation analysis, F = 59.349, *P* < 0.01

Of the 50 neonates with HIE, 42 (84.0%) had abnormal aEEG, and eight (16.0%) had normal aEEG. Serum 1-day NSE levels were not different among the groups (*P* = 0.929), but serum 3-day NSE levels increased with worsening neurodevelopment outcomes at 18 months of age (normal: 20.52 ± 6.42 μg/L vs. moderate: 39.82 ± 5.92 μg/L vs. severe: 44.60 ± 9.01 μg/L, *P* < 0.001; *P* < 0.01 normal vs. moderate, *P* < 0.01 normal vs. severe, *P* = 0.147 moderate vs. severe; 22 were above the cutoff value and 28 were below) (Table 1 and Fig. 2A). The MRI findings at 4–7 days after birth were significantly different among the three groups (*P* < 0.001) (Table 1).

### The predictive values of aEEG, 3-day NSE levels, and brain MRI for neurodevelopmental outcomes

As shown in Table 2, aEEG had high sensitivity (100%) but poor specificity (25.00%) for the neurodevelopmental outcomes at 18 months. MRI had good sensitivity (94.44%) and fair specificity (71.88%), while 3-day NSE had high sensitivity (100%) and specificity (87.50%). 3-day NSE levels were evaluated by ROC curve analysis, and the cut-off value that maximized sensitivity and specificity for the moderate or severe developmental delay was 27.3 μg/L (Fig. 2B). The combination of the three variables (high NSE, abnormal aEEG, and MRI abnormalities together) had a sensitivity of 100%, specificity of 97.70%, PPV of 98.25%, and NPV of 99.98% (Table 2).

**Table 2:** Predictive values of aEEG, MRI, and 3-day NSE for neurodevelopment outcomes (*n*=50)

Measure	Sensitivity	Specificity	PPV	NPV
aEEG-abnormal	100%	25.00%	42.86%	100.00%
MRI	94.44%	71.88%	65.38%	95.83%
3-day NSE	100%	87.50%	81.82%	100.00%
MRI + aEEG	94.45%	79.98%	66.56%	96.35%
aEEG+ MRI+ 3-day NSE	100%	97.70%	98.25%	99.98%

Sensitivity, specificity, PPV, and NPV were presented as percentages. *aEEG* Amplitude-integrated electroencephalography, *MRI* Magnetic resonance imaging, *3-day NSE* NSE level of 3 days after birth, *PPV* Positive predictive value, *NPV* Negative predictive value

## Discussion

Neonatal HIE is an important cause of mortality and morbidity [1, 2], but effective indicators for the early diagnosis of brain injury after asphyxia and prognosis are lacking [15–17]. Therefore, this study aimed to examine the predictive value of serum NSE, aEEG, and MRI, alone and in combination, for the neurological outcomes in neonates with HIE. The results suggest that the combination of MRI, aEEG and 3-day NSE can predict the neurological prognosis of newborns with HIE. The combination of the three methods can improve the predictive ability for long-term neurobehavioral prognosis.

Previous studies examined the value of the Sarnat score that could be used to determine the prognosis of the infants; Polat et al. [42] and Liauw et al. [43] showed that the PPV and NPV of the Sarnat staging for the adverse outcome (disability and death) were 52 and 100%, while the PPV and NPV were respectively 45 and 0% in children with HIE grade II [43]. In the present study, the Sarnat staging had difficulty in assessing neurodevelopment at 18 months due to the multifactor dependency of HIE, as supported by the above studies. Therefore, more reliable methods are needed. The Thompson score has been shown to be of prognostic value in infants with HIE [44] but was not assessed in this study. In this study, HIE grading was based on Sarnat grading but could be affected by a variety of clinical factors. Sarnat grading is not good in predicting long-term outcomes, and this study discusses other predictive indicators. This is consistent with Shankaran et al. [45].

Elevated NSE levels have been reported after stroke, brain injury, cardiac surgery, cardiopulmonary arrest, and perinatal asphyxia [46–48]. Kunda et al. [49] showed that important increases in NSE levels could be observed after seizures in patients with temporal lobe epilepsy, suggesting neuronal damage, but the cause-to-effect relationship could not be established. Lee et al. [50] suggested that NSE levels could be used for the differential diagnosis of seizures over syncope. In addition, Sheik et al. [51] showed that NSE is elevated in patients with acute diseases of the central nervous system, and that it was elevated in patients with seizures vs. those without. Therefore, NSE is associated with brain damage and seizures, but whether NSE levels increases before or after seizures remain to be determined.

HIE is a fetal or neonatal brain injury caused by decreased or suspended cerebral blood flow, with partial or complete asphyxia resulting from perinatal hypoxia. At the cellular level, decreased blood flow and oxygen delivery initiates a series of harmful biochemical reactions. Oxygen consumption interferes with oxidative phosphorylation, which leads to the conversion to anaerobic metabolism, rapid depletion of high-energy phosphate reserves, accumulation of lactic acid, and failure to maintain cellular function [1, 2, 4]. Cell membranes are damaged, and intracellular components of neurons, including NSE, are released into the cerebrospinal fluid and blood. In this study, increased NSE levels were observed at 3 days in neonates who eventually showed neurodevelopmental delay. It is supported by studies that suggested the value of NSE for HIE [52, 53]. Recent studies in asphyxiated neonates have correlated NSE with developmental outcomes, but the conclusions were conflicting because of different detection timing because it takes some time for the damaged cells to die and release their content [54], supporting the significant difference at 3 days but not at 1 day in the present study. Kecskes et al. [55] also reported that NSE levels at 3 days had the best predictive value. Still, it is unknown whether the higher NSE levels at 3 days compared with 1 day is the result of delayed NSE release in the blood compartment or the result of the ongoing injury process. This will have to be examined in future studies.

The abnormal electrical activity in brain regions can be diagnosed by aEEG. Nakamura et al. [56] reported that electrical changes could occur in damaged brain regions within 10–20 min after a hypoxic-ischemic attack, which can be detected by aEEG monitoring. The present study suggested that a persistent abnormal aEEG over 4 h and within 6 h after birth was associated with adverse neurodevelopmental outcomes. Still, the PPV of 6-h aEEG was poor (PPV of 42.9%), and good outcomes might occur despite an abnormal aEEG. Therefore, an abnormal aEEG within 6 h after birth might have no clinical value if it is used alone. Conversely, a normal 6-h aEEG has a good NPV (100%). These results are supported by a previous study [57], but Vasiljevic et al. [58] showed higher specificity and PPV than in the present study. Their study showed the pattern of abnormal aEEG correlated with the severity of HIE (*P* < 0.0001) and subsequent neurodevelopmental outcome (*P* < 0.001) [58]. They found that abnormal aEEG background patterns exhibited superior prediction of abnormal developmental outcomes at 12 months of age. Still, the discrepancies between the present and previous studies could be due to differences in monitoring time (within the first 72 h of life in their case [58]). aEEG is a useful tool for the monitoring and detection of seizures [59]. In neonates with seizures, more than two seizures detected by aEEG is predictive of progression to early-onset epileptic encephalopathy [60]. Still, the exact relationship between seizures and aEEG abnormalities could not be determined in the present study. Future studies will have to examine the exact role of seizures, duration of seizures, and seizure burden in infants with HIE.

Conventional MRI is an essential tool for assessing the neonatal brain injury and degree of injury, but sometimes T1- and T2-weighted images can appear normal during the few days after birth or show only subtle findings that might be difficult to interpret or to distinguish from normal maturational phenomena in the neonatal brain. The most appropriate time for examination is 4–7 days after birth [35, 36]. The present study showed that in term infants with HIE, conventional MRI detection 4–7 days after birth can be used for the relatively accurate prediction of neurodevelopment outcomes at 18 months old (sensitivity, specificity, PPV, and NPV, respectively were 94.44, 71.88, 65.38, and 95.83%).

Still, whether a combination of NSE, MRI, and EEG could improve the prognostication of HIE remains to be examined. Indeed, Leon-Lozano et al. [21] and Shim et al. [22] recently reported NSE levels, MRI, and aEEG (all assessed within the same study) on the outcomes of HIE, but without combining the different modalities. A previous study published by Ezgu et al. in 2002 [23] suggested that the combination of NSE, MRI, and EEG could be used to determine the prognosis of HIE, but they measured NSE in the cerebrospinal fluid, which is less practical than from the blood. In the present study, consistent relationships between the outcomes at 18 months and the injury degree on conventional MRI, aEEG results, and serum NSE levels of 3 days after birth (r_s_ = 0.719, *P* < 0.001) were observed. When dichotomizing the NSE levels based on the best cut-off value and when considering MRI and aEEG as normal/abnormal, combining the three test results in better sensitivity, specificity, PPV, and NPV than for each test alone. This possible predictive tool should be validated in a larger group of patients.

In this study, 27% of the children with mild HIE had a severe delay. Most previous studies used the traditional clinical Sarnat grading system, in which infants with mild Sarnat grading had a good prognosis and no long-term disability. For this reason, many studies did not carry out a systematic examination and long-term follow-up on neonates with mild HIE. Still, more and more studies are finding that they can experience major disability, and when followed up to 18 months, about 25% of infants with mild HIE have abnormal outcomes, defined as death or movement and/or developmental delay [61, 62]. Nevertheless, it is not clear why a minor injury would lead to such severe adverse outcomes. It might be that early hypoxia injury will change, worsen, and expand over time. Since a significant proportion of children with mild encephalopathy have been reported to have significant long-term neurological sequelae, more measures are needed to evaluate brain injury and better predict prognosis in addition to Sarnat grading.

There are limitations of this study. The sample size was small and from a single center. Long-term outcomes were not followed. A larger sample size, multiple centers, and longer clinical follow-up are needed to confirm the findings. In addition, additional indicators of electrophysiological and biochemical changes of neurons after hypoxia and ischemia should be explored. Of note, the MRI examinations were read by a general radiologist, not a pediatric radiologist or a neuroradiologist. Due to copyright issues, Bayley III cannot be translated, standardized, and validated in Chinese for the time being; therefore, Bayley II was used. Finally, in this study,the mortality among moderate/severe HIE infants was much lower than seen in the RCTs of therapeutic hypothermia and in subsequent prospective studies, part of the reason is that the special and complex doctor-patient relationship in China. When there is subjective evaluation for disease severity, Chinese doctors tend to classify them as severe cases, which is relatively common and may be different from other countries, may have had some effect on the results.

In conclusion, in the earlier stages after birth, aEEG, MRI, and 3-day NSE levels can be used to predict the 18-month neurodevelopmental outcomes of children with HIE not treated with hypothermia treatment. These tests might have a use in predicting prognosis in full-term infants with HIE, providing them with more accurate management strategies.

## Data Availability

The datasets generated and/or analysed during the current study are not publicly available due to the prospective study, data are incomplete and further studies are needed, but are available from the corresponding author on reasonable request.

## Abbreviations

HIE: Hypoxic-ischemic encephalopathy
NSE: Neuron-specific enolase
aEEG: Amplitude-integrated electroencephalography
MRI: Magnetic resonance imaging

## References

[1] Executive summary: Neonatal encephalopathy and neurologic outcome, second edition (2014). Report of the American College of Obstetricians and Gynecologists' task force on neonatal encephalopathy. Obstet Gynecol.

[2] Wachtel EV, Hendricks-Munoz KD (2011). Current management of the infant who presents with neonatal encephalopathy. Curr Probl Pediatr Adolesc Health Care.

[3] Volpe JJ (2001). Hypoxic-ischemic encephalopathy in neurology of the newborn.

[4] Douglas-Escobar M, Weiss MD (2015). Hypoxic-ischemic encephalopathy: a review for the clinician. JAMA Pediatr.

[5] Weitzdoerfer R, Gerstl N, Pollak D, Hoeger H, Dreher W, Lubec G (2004). Long-term influence of perinatal asphyxia on the social behavior in aging rats. Gerontology..

[6] Martinez-Biarge M, Diez-Sebastian J, Kapellou O, Gindner D, Allsop JM, Rutherford MA, Cowan FM (2011). Predicting motor outcome and death in term hypoxic-ischemic encephalopathy. Neurology..

[7] Efstathiou N, Theodoridis G, Sarafidis K (2017). Understanding neonatal hypoxic-ischemic encephalopathy with metabolomics. Hippokratia..

[8] Bano S, Chaudhary V, Garga UC (2017). Neonatal hypoxic-ischemic encephalopathy: a radiological review. J Pediatr Neurosci.

[9] Haataja L, Mercuri E, Guzzetta A, Rutherford M, Counsell S, Flavia Frisone M, Cioni G, Cowan F, Dubowitz L (2001). Neurologic examination in infants with hypoxic-ischemic encephalopathy at age 9 to 14 months: use of optimality scores and correlation with magnetic resonance imaging findings. J Pediatr.

[10] Kaufman SA, Miller SP, Ferriero DM, Glidden DH, Barkovich AJ, Partridge JC (2003). Encephalopathy as a predictor of magnetic resonance imaging abnormalities in asphyxiated newborns. Pediatr Neurol.

[11] Mrelashvili A, Russ JB, Ferriero DM, Wusthoff CJ (2020). The Sarnat score for neonatal encephalopathy: looking back and moving forward. Pediatr Res.

[12] Allen KA, Brandon DH (2011). Hypoxic ischemic encephalopathy: pathophysiology and experimental treatments. Newborn Infant Nurs Rev.

[13] Chao CP, Zaleski CG, Patton AC (2006). Neonatal hypoxic-ischemic encephalopathy: multimodality imaging findings. Radiographics..

[14] Ghei SK, Zan E, Nathan JE, Choudhri A, Tekes A, Huisman TA, Izbudak I (2014). MR imaging of hypoxic-ischemic injury in term neonates: pearls and pitfalls. Radiographics..

[15] El-Ayouty M, Abdel-Hady H, El-Mogy S, Zaghlol H, El-Beltagy M, Aly H (2007). Relationship between electroencephalography and magnetic resonance imaging findings after hypoxic-ischemic encephalopathy at term. Am J Perinatol.

[16] Forbes KP, Pipe JG, Bird R (2000). Neonatal hypoxic-ischemic encephalopathy: detection with diffusion-weighted MR imaging. AJNR Am J Neuroradiol.

[17] Fan G, Wu Z, Chen L, Guo Q, Ye B, Mao J (2003). Hypoxia-ischemic encephalopathy in full-term neonate: correlation proton MR spectroscopy with MR imaging. Eur J Radiol.

[18] Douglas-Escobar M, Weiss MD (2012). Biomarkers of hypoxic-ischemic encephalopathy in newborns. Front Neurol.

[19] Celtik C, Acunas B, Oner N, Pala O (2004). Neuron-specific enolase as a marker of the severity and outcome of hypoxic ischemic encephalopathy. Brain and Development.

[20] Sun J, Li J, Cheng G, Sha B, Zhou W (2012). Effects of hypothermia on NSE and S-100 protein levels in CSF in neonates following hypoxic/ischaemic brain damage. Acta Paediatr.

[21] Leon-Lozano MZ, Arnaez J, Valls A, Arca G, Agut T, Alarcon A, Garcia-Alix A (2020). Cerebrospinal fluid levels of neuron-specific enolase predict the severity of brain damage in newborns with neonatal hypoxic-ischemic encephalopathy treated with hypothermia. PLoS One.

[22] Shim GH (2021). Which factors predict outcomes of neonates with hypoxic-ischemic encephalopathy following therapeutic hypothermia?. Clin Exp Pediatr.

[23] Ezgu FS, Atalay Y, Gucuyener K, Tunc S, Koc E, Ergenekon E, Tiras U (2002). Neuron-specific enolase levels and neuroimaging in asphyxiated term newborns. J Child Neurol.

[24] Schupper A, Almashanu S, Coster D, Keidar R, Betser M, Sagiv N, Bassan H (2021). Metabolic biomarkers of small and large for gestational age newborns. Early Hum Dev.

[25] Glover Williams A, Odd D (2020). Investigating the association between post-term birth and long term cognitive, developmental and educational impacts: a systematic review and Meta-analysis. J Matern Fetal Neonatal Med.

[26] Rolschau AH, Olesen AW, Obel C, Olsen J, Wu CS, Kofoed PE (2020). Cerebral disorders in the first 7 years of life in children born post-term: a cohort study. BMC Pediatr.

[27] Wang W, Sun K, Chang L, Shen K, Li Q, Du L, Mu D (2018). Pediatrics.

[28] Use and abuse of the Apgar score (1996). Committee on fetus and newborn, American Academy of Pediatrics, and committee on obstetric practice, American College of Obstetricians and Gynecologists. Pediatrics..

[29] Watson RS, Crow SS, Hartman ME, Lacroix J, Odetola FO (2017). Epidemiology and outcomes of pediatric multiple organ dysfunction syndrome. Pediatr Crit Care Med.

[30] Azzopardi D, group Ts. (2014). Predictive value of the amplitude integrated EEG in infants with hypoxic ischaemic encephalopathy: data from a randomised trial of therapeutic hypothermia. Arch Dis Child Fetal Neonatal Ed.

[31] Hellstrom-Westas L, Rosen I, Svenningsen NW (1995). Predictive value of early continuous amplitude integrated EEG recordings on outcome after severe birth asphyxia in full term infants. Arch Dis Child Fetal Neonatal Ed.

[32] Hellstrom-Westas L, Rosen I (2006). Continuous brain-function monitoring: state of the art in clinical practice. Semin Fetal Neonatal Med.

[33] Thoresen M, Hellstrom-Westas L, Liu X, de Vries LS (2010). Effect of hypothermia on amplitude-integrated electroencephalogram in infants with asphyxia. Pediatrics..

[34] Trivedi SB, Vesoulis ZA, Rao R, Liao SM, Shimony JS, McKinstry RC, Mathur AM (2017). A validated clinical MRI injury scoring system in neonatal hypoxic-ischemic encephalopathy. Pediatr Radiol.

[35] Rutherford MA, Pennock JM, Schwieso JE, Cowan FM, Dubowitz LM (1995). Hypoxic ischaemic encephalopathy: early magnetic resonance imaging findings and their evolution. Neuropediatrics..

[36] Barkovich AJ, Miller SP, Bartha A, Newton N, Hamrick SE, Mukherjee P, Glenn OA, Xu D, Partridge JC, Ferriero DM (2006). MR imaging, MR spectroscopy, and diffusion tensor imaging of sequential studies in neonates with encephalopathy. AJNR Am J Neuroradiol.

[37] Barkovich AJ, Hajnal BL, Vigneron D, Sola A, Partridge JC, Allen F, Ferriero DM (1998). Prediction of neuromotor outcome in perinatal asphyxia: evaluation of MR scoring systems. AJNR Am J Neuroradiol.

[38] Gagnon SG, Nagle RJ (2016). Comparison of the revised and original versions of the Bayley scales of infant development. School Psychol Intl.

[39] van Schie PE, Becher JG, Dallmeijer AJ, Barkhof F, Van Weissenbruch MM, Vermeulen RJ (2010). Motor testing at 1 year improves the prediction of motor and mental outcome at 2 years after perinatal hypoxic-ischaemic encephalopathy. Dev Med Child Neurol.

[40] Bayley N (1993). Bayley scales of infant development.

[41] Jiang CM, Yang YH, Chen LQ, Shuai XH, Lu H, Xiang JH, Liu ZL, Zhu YX, Xu RY, Zhu DR (2015). Early amplitude-integrated EEG monitoring 6 h after birth predicts long-term neurodevelopment of asphyxiated late preterm infants. Eur J Pediatr.

[42] Polat M, Simsek A, Tansug N, Sezer RG, Ozkol M, Baspinar P, Tekgul H (2013). Prediction of neurodevelopmental outcome in term neonates with hypoxic-ischemic encephalopathy. Eur J Paediatr Neurol.

[43] Liauw L, van der Grond J, van den Berg-Huysmans AA, Laan LA, van Buchem MA, van Wezel-Meijler G (2008). Is there a way to predict outcome in (near) term neonates with hypoxic-ischemic encephalopathy based on MR imaging?. AJNR Am J Neuroradiol.

[44] Thompson CM, Puterman AS, Linley LL, Hann FM, van der Elst CW, Molteno CD, Malan AF (1997). The value of a scoring system for hypoxic ischaemic encephalopathy in predicting neurodevelopmental outcome. Acta Paediatr.

[45] Shankaran S, Laptook AR, Ehrenkranz RA, Tyson JE, McDonald SA, Donovan EF, Fanaroff AA, Poole WK, Wright LL, Higgins RD (2005). Whole-body hypothermia for neonates with hypoxic-ischemic encephalopathy. N Engl J Med.

[46] Freeman WD, Chiota NA (2011). Neuron-specific enolase correlates with other prognostic markers after cardiac arrest. Neurology..

[47] Hu Y, Meng R, Zhang X, Guo L, Li S, Wu Y, Duan J, Ding Y, Ji X (2018). Serum neuron specific enolase may be a marker to predict the severity and outcome of cerebral venous thrombosis. J Neurol.

[48] Maggiotto LV, Sondhi M, Shin BC, Garg M, Devaskar SU (2019). Circulating blood cellular glucose transporters - surrogate biomarkers for neonatal hypoxic-ischemic encephalopathy assessed by novel scoring systems. Mol Genet Metab.

[49] Kunda S, LaFrance-Corey RG, Khadjevand F, Worrell GA, Howe CL (2019). Systemic evidence of acute seizure-associated elevation in serum neuronal injury biomarker in patients with temporal lobe epilepsy. Acta Epileptol.

[50] Lee SY, Choi YC, Kim JH, Kim WJ (2010). Serum neuron-specific enolase level as a biomarker in differential diagnosis of seizure and syncope. J Neurol.

[51] Shaik AJ, Reddy K, Mohammed N, Tandra SR, Rukmini Mridula K, Baba KS (2019). Neuron specific enolase as a marker of seizure related neuronal injury. Neurochem Int.

[52] Verdu Perez A, Falero MP, Arroyos A, Estevez F, Felix V, Lopez Y, Pantoja A, Ureta A (2001). Blood neuronal specific enolase in newborns with perinatal asphyxia. Rev Neurol.

[53] Berger RP, Pak BJ, Kolesnikova MD, Fromkin J, Saladino R, Herman BE, Pierce MC, Englert D, Smith PT, Kochanek PM (2017). Derivation and validation of a serum biomarker panel to identify infants with acute intracranial hemorrhage. JAMA Pediatr.

[54] Serhan CN, de la Rosa X, Jouvene C (2019). Novel mediators and mechanisms in the resolution of infectious inflammation: evidence for vagus regulation. J Intern Med.

[55] Kecskes Z, Dunster KR, Colditz PB (2005). NSE and S100 after hypoxia in the newborn pig. Pediatr Res.

[56] Nakamura S, Kusaka T, Koyano K, Miki T, Ueno M, Jinnai W, Yasuda S, Nakamura M, Okada H, Isobe K (2014). Relationship between early changes in cerebral blood volume and electrocortical activity after hypoxic-ischemic insult in newborn piglets. Brain and Development.

[57] Chandrasekaran M, Chaban B, Montaldo P, Thayyil S (2017). Predictive value of amplitude-integrated EEG (aEEG) after rescue hypothermic neuroprotection for hypoxic ischemic encephalopathy: a meta-analysis. J Perinatol.

[58] Vasiljevic B, Maglajlic-Djukic S, Gojnic M (2012). The prognostic value of amplitude-integrated electroencephalography in neonates with hypoxic-ischemic encephalopathy. Vojnosanit Pregl.

[59] Glass HC, Wusthoff CJ, Shellhaas RA (2013). Amplitude-integrated electro-encephalography: the child neurologist's perspective. J Child Neurol.

[60] Liu LL, Hou XL, Zhang DD, Sun GY, Zhou CL, Jiang Y, Tang ZZ, Zhang R, Cui Y (2017). Clinical manifestations and amplitude-integrated encephalogram in neonates with early-onset epileptic encephalopathy. Chin Med J.

[61] Conway JM, Walsh BH, Boylan GB, Murray DM (2018). Mild hypoxic ischaemic encephalopathy and long term neurodevelopmental outcome - a systematic review. Early Hum Dev.

[62] Murray DM, O'Connor CM, Ryan CA, Korotchikova I, Boylan GB. Early EEG Grade and Outcome at 5 Years After Mild Neonatal Hypoxic Ischemic Encephalopathy. Pediatrics. 2016;138(4):1–6. 10.1542/peds.2016-0659 27650049

